# The Promise and Challenges of Developing miRNA-Based Therapeutics for Parkinson’s Disease

**DOI:** 10.3390/cells9040841

**Published:** 2020-03-31

**Authors:** Simoneide S. Titze-de-Almeida, Cristina Soto-Sánchez, Eduardo Fernandez, James B. Koprich, Jonathan M. Brotchie, Ricardo Titze-de-Almeida

**Affiliations:** 1 Technology for Gene Therapy Laboratory, Central Institute of Sciences, FAV, University of Brasilia, Brasília 70910-900, Brazil; simoneide.silva@gmail.com; 2 Neuroprosthetics and Visual Rehabilitation Research Unit, Bioengineering Institute, Miguel Hernández University, 03202 Alicante, Spain; csoto@goumh.umh.es (C.S.-S.); e.fernandez@umh.es (E.F.); 3 Biomedical Research Networking Center in Bioengineering, Biomaterials and Nanomedicine—CIBER-BBN, 28029 Madrid, Spain; 4 Krembil Neuroscience Centre, Toronto Western Hospital, University Health Network, Toronto, Ontario M5T 2S8, Canada; j.koprich@atuka.com (J.B.K.); j.brotchie@atuka.com (J.M.B.)

**Keywords:** Parkinson’s disease, alpha-synuclein, microRNA, RNA interference, gene silence, RNAi therapeutic

## Abstract

MicroRNAs (miRNAs) are small double-stranded RNAs that exert a fine-tuning sequence-specific regulation of cell transcriptome. While one unique miRNA regulates hundreds of mRNAs, each mRNA molecule is commonly regulated by various miRNAs that bind to complementary sequences at 3’-untranslated regions for triggering the mechanism of RNA interference. Unfortunately, dysregulated miRNAs play critical roles in many disorders, including Parkinson’s disease (PD), the second most prevalent neurodegenerative disease in the world. Treatment of this slowly, progressive, and yet incurable pathology challenges neurologists. In addition to L-DOPA that restores dopaminergic transmission and ameliorate motor signs (i.e., bradykinesia, rigidity, tremors), patients commonly receive medication for mood disorders and autonomic dysfunctions. However, the effectiveness of L-DOPA declines over time, and the L-DOPA-induced dyskinesias commonly appear and become highly disabling. The discovery of more effective therapies capable of slowing disease progression –a neuroprotective agent–remains a critical need in PD. The present review focus on miRNAs as promising drug targets for PD, examining their role in underlying mechanisms of the disease, the strategies for controlling aberrant expressions, and, finally, the current technologies for translating these small molecules from bench to clinics.

## 1. The Underlying Mechanisms of Parkinson’s Disease (PD)

Parkinson’s disease (PD) is a neurodegenerative disorder affecting over 6 million people globally and with rising incidence as the aged population continues to grow [1]. PD is typically classified as a movement disorder with a triad of bradykinesia, rigidity and tremor [2]. The major pathology contributing to the generation of these motor symptoms is degeneration of the dopaminergic nigrostriatal pathway [3]. Indeed, dopamine replacement therapy has provided the basis of symptomatic therapy for more than half a century. However, the benefits of symptomatic therapies are limited by the emergence of side effects and the loss of efficacy over time and the fact that they do not treat all symptoms of PD equally well [4]. These limitations emerge in part because of plastic changes in brain circuits in response to long term treatment, in part because of the insidious progression of the underlying disease and partly because not all symptoms of PD are driven by nigrostriatal dysfunction [5]. With respect to the latter, in addition to the above-mentioned motor symptoms, PD is also associated with a wide range of non-motor symptoms, and most of these remain significant unmet medical needs [6]. Such non-motor symptoms include cognitive and behavioral problems (executive dysfunction [7], psychosis [8], apathy [9], mood disorders) [10], autonomic failure (e.g., urinary, bladder disturbances and constipation) [11], loss of sense of smell [12], sleep disturbance [13], and pain [14] and involve neural circuits in addition to the nigrostriatal pathway.

Irrespective of etiology, genetic or sporadic, most PD cases show alpha-synuclein (α-Syn)-related pathology marked by the presence of Lewy bodies and Lewy neurites [15]. While α-Syn accumulation is found within the nigrostriatal pathway and accompanies the degenerative process, this synucleinopathy is actually widespread throughout the brain and peripheral nervous system including in regions that likely contribute to non-motor symptoms, e.g., early in the disease in the enteric plexus, dorsal nucleus of the vagal nerve, locus coeruleus, dorsal raphe nucleus and, late in the disease, advancing into the cerebral cortex [5,16,17].

Thus, current symptomatic therapies do not provide effective long-term control of symptoms or underlying disease and do little to deal with problems emerging outside of the nigrostriatal pathway. There is a need for disease-modifying therapies for PD and, ideally, those that would slow neurodegeneration and functional pathology not only in the nigrostriatal pathway but also in other regions of the nervous system contributing to non-motor symptoms. This need is, so far, unmet.

The finding that α-Syn pathology is a hallmark of PD, whatever the etiology, suggests that, despite likely divergence of upstream initiating events, there is a common downstream convergence leading to the involvement of α-Syn as a key player in disease pathogenesis. Many mechanisms of pathology have been proposed, and multiple targets identified to potentially manipulate these. These targets have, to varying degrees, been validated in cell-based systems, animal models and PD post-mortem material. Such targets include, of course, α-Syn, but extend to a panoply of mechanisms that might impact upon α-Syn, either in all or subsets of the PD population, these include oxidative stress [18], LRRK2 [19], apoptosis [20], neuroinflammation [21], misfolding proteins [22], age [2], melanin [23] and iron accumulation [24]. The most attractive of these targets also have support from genetic studies, and include LRRK2 [25], parkin, PINK1 [26] and GBA [27]. MicroRNAs are also emerging targets that have gained increased attention in neurodegenerative disorders, including PD [28]. These small RNAs play relevant roles in regulating cell transcriptome by RNA interference, but they can also contribute to disease pathogenesis if aberrantly expressed [29,30]. In such sense, a dysregulated expression of a specific microRNA can be set up to the physiological level by synthetic oligos for disrupting critical mechanisms of disease pathogenesis [31,32].

This review examines the role of microRNAs (miRNAs) in underlying mechanisms of PD and updates on advances in technologies that improve efficacy and delivery of small RNAs, paving the way toward a miRNA-based neuroprotective therapy.

## 2. MicroRNA Biogenesis

**Figure 1:**
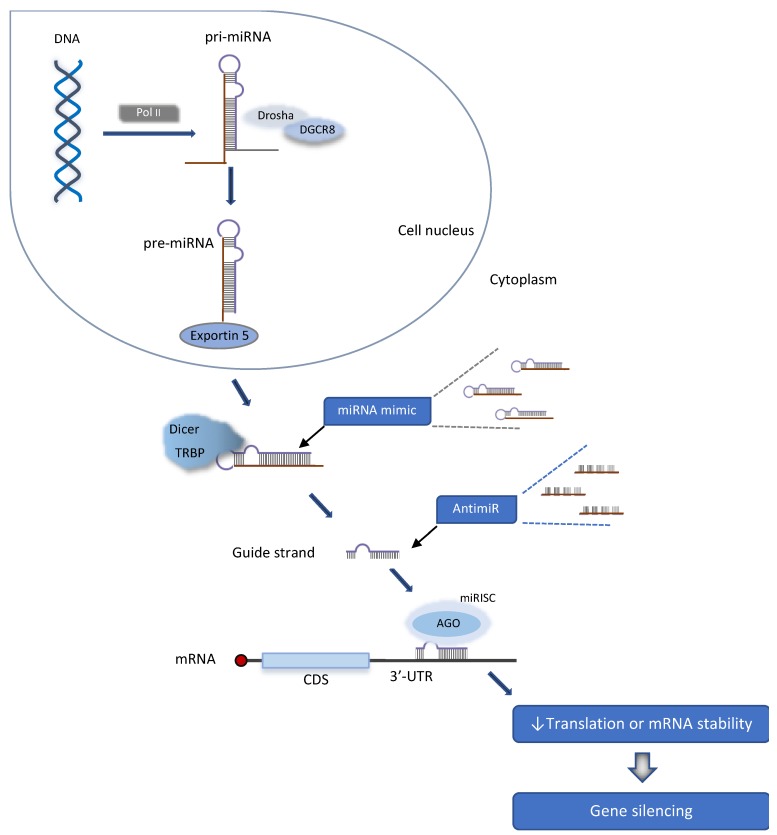
Biological synthesis of endogenous microRNAs (miRNAs) and modulation by synthetic oligonucleotides. miRNAs are coded in mammalian DNA genes and transcribed by RNA polymerase II (Pol II) to form the primary miRNA (pri-miRNA). This long RNA receives the first processing by Drosha and DGCR8 enzymes in cell nucleus, with remotion of nucleotides outside the hairpin. The resulting miRNA precursor (pre-miRNA) moves to cytoplasm carried by Exportin 5. Dicer and TRBP enzymes execute the second round of processing, resulting in miRNA duplexes with 18–25 nucleotides. Sense strand is removed. The guide (or antisense) strand is the mature miRNA that will guide the RISC complex (miRISC) to target mRNAs bearing partially complementary sequences in 3’-UTR region. Silencing of miRNA-targeted mRNAs occurs through translational repression or degradation. AGO—Argonaute 2; CDS—Coding sequence region of mRNA; 3’-UTR-3’ untranslated region; DGCR8—DiGeorge syndrome critical region gene 8; Dicer—a ribonuclease enzyme; Drosha—a ribonuclease enzyme; miRISC—RISC complex associated with a miRNA; Pol II—RNA polymerase II; pre-miRNA—miRNA precursor; pri-miRNA—primary miRNA; RISC—RNA-induced silencing complex; TRBP—HIV-1 Trans-activation response (TAR) RNA-binding protein. Reprinted with permission from Titze-de-Almeida and Titze-de-Almeida 2018, with modifications [34].

MicroRNAs are small RNA molecules that commonly originate from a ‘canonical’ pathway in which the biosynthesis begin in the cell nucleus and extend to the cytoplasm (Figure 1) [33]. Two other sources of miRNAs exist; these molecules can be formed in cells from a ‘noncanonical’ pathway, and, in addition, they can be chemically synthesized for experimental assays as oligonucleotides that imitate the endogenous molecules, the miRNA mimics [33,34]. The Box 1 of Supplementary Material presents brief definitions of miRNA mimics and AntimiRs.

In the ‘canonical’ pathway, miRNA synthesis begins with a transcriptional process from the mammalian genome by RNA polymerase II (Pol II). A steam loop structure denominated primary miRNA (pri-miRNA) is formed in the cell nucleus. Two rounds of processing will subsequently occur. Drosha and DGCR8 enzymes cleave sequences outside the hairpin of the pri-miRNA, to form a precursor miRNA (pre-miRNA). Pre-miRNA are transported to the cytoplasm by exportin 5, and a second round of processing takes place. Dicer enzyme removes the loop of pre-miRNA to generate a mature miRNA duplex, with ≈ 22 nucleotides [33,35]. The miRNA duplex is incorporated into the RNA-induced silencing complex (miRISC) and the passenger strand is removed. The remaining guide strand loaded in the miRISC will identify and bind to complementary sequences in the 3’-untranslated (3’-UTR) region of target mRNAs by Watson-Crick base-pairing, thus allowing the attached miRISC to exert a target-specific RNA interference. Base pairing between miRNA and mRNA contains mismatches that prevent AGO2 from cleaving the target mRNA. The RNAi process thus occurs in consequence of translational repression at ribosomes and further mRNA degradation [33,34,35].

**Figure 2:**
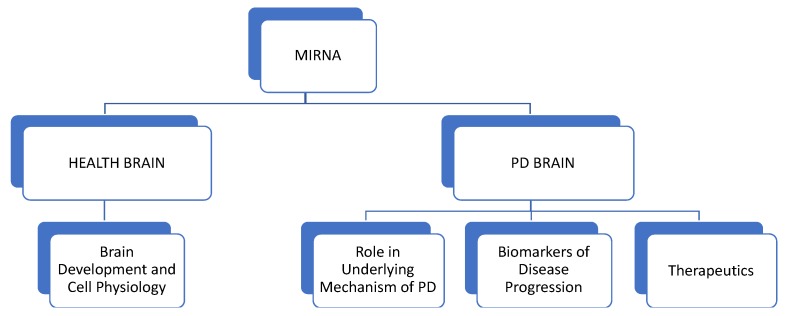
Schematic representation of miRNAs in the healthy and Parkinsonian brain. MicroRNAs expressed in the central nervous system contributes to brain development and cell physiology. Instead, aberrantly expressed miRNAs play a role in pathological mechanisms in a parkinsonian brain (PD brain). Translating to clinics, miRNAs are candidate biomarkers of disease progression and promising targets for miRNA-based therapeutics.

While microRNAs play a critical role in regulating cell mRNA levels in a healthy brain, they can assume a pathological function if aberrantly expressed, thereby contributing to the underlying mechanisms of PD (Table 1) [28]. In terms of disease management, dysregulated miRNAs are candidate molecules for diagnosis and prognosis of disease progression, and promising targets for drug development as well, as shown in Figure 2.

The following topic examines how dysregulated miRNAs contribute to synucleinopathy and neuroinflammation in PD, and may represent targets for a disease-modifying therapy.

## 3. miRNAs and Alpha Synuclein (α-Syn) Accumulation

α-Syn is expressed abundantly in the brain of healthy individuals and has gained increased attention as a component of the Lewy bodies in PD and other synucleinopathies [60,61,62,63]. This small protein of 140-amino acids plays a critical role in clustering synaptic vesicles at the presynaptic terminals [64,65,66]. α-Syn also regulates protein ubiquitination, chaperone activity, kinase-dependent pathways, and, lastly, the metabolism of dopamine [67,68,69,70]. Indeed, genome-wide association studies (GWAS) identified that α-Syn expression, glycosphingolipid biosynthesis, and the protein ubiquitination are pathways affected by single nucleotide polymorphisms (SNP) and a set of microRNAs differentially expressed in PD [71,72,73].

In healthy individuals, α-Syn is a soluble protein arranged in a stably-folded tetramer [74]. In PD, α-Syn forms deposits of insoluble protofibrils and fibrils with an antiparallel β-sheet structure of monomers, dimers, and misfolded oligomers [75,76,77]. α-Syn aggregates are located inside the Lewy bodies, present widely in critical brain regions and in peripheral body tissues [5,78,79]. The mechanism of widespread distribution of Lewy bodies might be a progressive cell-to-cell propagation of α-Syn aggregates, consistent with the hypothesis that PD is a prionic disease [78,80,81], whereby α-Syn templates allow protein propagation that drives a complex, multifactorial, and yet poorly understood neuropathology [82].

Mounting evidence supports that miRNAs are critical players for α-Syn accumulation in apoptotic neurons [83,84]. First, functional studies reveal specific miRNAs that are downregulated in PD, which may contribute to α-Syn accumulation and the consequent loss of dopaminergic neurons [28,29]. Second, the 3′-UTR region of α-Syn mRNA contains functional miRNA binding sites that are highly conserved among different species [85]. At least four distinct microRNAs directly downregulate α-Syn, miR-7, miR-153, miR34b/c, and miR-214, meaning that a change in their levels may lead to α-Syn accumulation in the cells [36,37,39,40].

MiR-7 well represents how an alteration in microRNA content directly affects a targeted mRNA. Patients with PD present reduced levels of miR-7 in brain regions related to disease neuropathology, especially the substantia nigra, and depletion of this miRNA is functionally related with α-Syn accumulation and the further neuron loss [36,86]. In addition to miR-7, miR-153 also recognizes sequences in 3′-UTR region of α-Syn, thereby operating additively to downregulate the gene. MiR-7 causes a stronger down-regulation over α-Syn compared to miR-153 [37]. Increased expression of miR-7 and miR-153 exert neuroprotective actions over dopaminergic cell types, as evidenced in the 1-methyl-4-phenylpyridinium (MPP+) in vitro model of PD [34,37,39].

Aberrant expression of miR-34c-5p also affects α-Syn deposition in PD brain tissues. This miRNA is depleted in amygdala, frontal cortex, substantia nigra and cerebellum of PD patients, as reported by Minones-Moyano and colleagues [55]. Depletion of miR-34c-5P begins in the premotor stages of the disease, thus we may speculate that a lack of this miRNA may have contributed to the slowly progressive loss of dopaminergic cells before the motor signs emerge. In addition, miR-34c-5P remains dysregulated later in the disease, thus having a potential role throughout disease progression [55]. A further report showed that miR-34b/c downregulates α-Syn by targeting to specific 3’-UTR sequence [40]. Conversely, the depletion of miR-34b/c leads to α-Syn accumulation with a subsequent reduction in viability of SH-SY5Y cells that also show oxidative stress, mitochondrial dysfunction, and reduction in DJ1 and Parkin expression [55].

Finally, three other microRNAs are involved in α-Syn regulation, exerting a direct regulation by binding to its 3’-UTR region (miR-214), or an indirect effect, as occurs for Let-7 and miR-16-1 [41,43,87].

Let-7 regulates α-Syn via the autophagy-lysosome network, a pathway that executes α-Syn clearance under physiological conditions [87,88,89,90]. In a *Caenorhabditis elegans* model of PD, depletion of let-7 led to α-Syn accumulation. The change was related to increased levels of lgg-1 and atg-13 and consequent degradation of cellular components and autophagy-related genes, respectively [87,91,92,93]. miR-16-1 has several binding sites in the 3′-UTR region of HSP70. Growing evidence supports the concept that dysregulated chaperones, especially Hsp70, are involved in PD pathogenesis [94,95]. In such a sense, Hsp70 plays a role in aggregation and cytotoxicity of α-Syn in PD, as confirmed by functional studies and miR-16-1 transfections in SH-SY5Y cells [43].

## 4. microRNAs and Neuroinflammation

MicroRNAs are important modulators of neuroinflammation, a process found in brain regions involved in PD pathogenesis [96,97]. First, the process of neuroinflammation develops in parallel to and contributes to the death of neuron cells [98,99]. Affected areas present a higher content of activated microglia and astrocytic cells, both findings reported in parkinsonian animals and PD patients [21,100,101,102,103]. In agreement with this role, pharmacological inhibitors of microglia activation prevent the loss of nigral neurons in animal models of PD [104,105,106,107].

Neuroinflammation is affected by, and affects, α-Syn accumulation in a bidirectional feedback loop. First, α-Syn aggregates activate microglial cells [108]. Activated microglia, in turn, lead to abnormal handling of α-Syn in neurons that further induces a pro-inflammatory process, with increased levels of cytokine, nitric oxide, and reactive oxygen species [109]. Accumulation of α-Syn in cerebral neurons correlates with the presence of HLA-DR (human homolog of MHCII) expressed by microglia and, in addition, with deposits of immunoglobulin G (IgG) in neuronal cells [110,111]. Moreover, the levels of pro-inflammatory cytokines IL1-β (interleukin-1 β), interleukin-2 (IL-2), interleukin-6 (IL-6), interferon-γ (IFN-γ) and tumor necrosis factor alpha (TNF-α) are increased in the nigrostriatal dopaminergic system and peripheral nerves of PD patients [112,113,114,115,116,117].

TNF-α, a critical regulator of inflammatory responses, was found elevated in the blood, CSF, and striatum of PD patients [112,114,115]. This cytokine can damage SH-SY5Y cells and, in addition, increase their vulnerability to 1-methyl-4-phenyl-1,2,3,6-tetrahydropyridine (MPTP), 6-OHDA and rotenone [49]. TNF-α also regulates miRNAs that target proteins of the mitochondrial complex-I and V. Inhibition of miR-155—a microRNA that targets ATP5G3 (a subunit of F1-ATP synthase)—can attenuate the death of SH-SY5Y cells induced by TNF-α [49].

miR-7 has also been implicated in neuroinflammatory process related to the loss of nigrostriatal cells [36,47]. The gene NRLP3 (inflammasome nod-like receptor protein 3) expressed in microglial cells is down-regulated by miR-7 [47]. This work showed that injections of miR-7 mimics into mouse striatum can suppress NLRP3 inflammasome activation and reduce the loss of dopaminergic cells in MPTP-injured mice model of PD. Effects of miR-7 against neuroinflammation were corroborated by other studies. Thus, miR-7 knocked-down RelA, a component of NF-κB—a transcriptional factor that regulates genes involved in inflammation and cellular death. Indeed, silencing of RelA induced by miR-7 attenuates the damage of MPP+ to SH-SY5Ycells [38,118,119,120,121].

## 5. Biotechnology for Moving microRNAs from the Bench to Clinics

### 5.1. Steps to Develop and Evaluate miRNA-Based Drugs

A biotechnological platform aimed to develop miRNA-based therapies broadly follows the stages applied to classic small molecule drug as shown (see Box 2 of the Supplementary Material); Step 1—Discovery and Development; Step 2—Preclinical Research; Step 3—Clinical Research (Phases 1–3 clinical trials); Step 4—FDA Review; Step 5—Post-Market Safety monitoring (Phase 4) [122].

Currently, ongoing clinical trials are employing miRNA- mimics or inhibitors that act on distinct targets and address diseases with different pathogenesis, revealing the flexibility of this biotechnology. Examples of microRNA-based therapeutics in clinical testing are: hepatitis C (AntimiR-122), type 2 diabetes and non-alcoholic fatty liver diseases (AntimiR-103/107), T-cell lymphoma and leukemias, mycosis fungoides (AntimiR-155), scleroderma (miR-29 mimic), mesothelioma and lung cancer (miR-16 mimic), wound healing and heart failure (miR-92), keloids and fibrous scar tissue formation (miR-92), and Alport syndrome (miR-21) [123,124,125,126].

### 5.2. Increased Duration of Effects and Site-Specific Delivery: Two Critical Issues for RNAi-Based Drugs for Brain Diseases

RNAi-based drugs have shown therapeutic benefits in chronic diseases outside the nervous system, as demonstrated by the first FDA-approved siRNA named patisiran [127]. At doses of 0.3 mg/Kg administered intravenously every three weeks, patisiran caused a gene knockdown stable for at least two years [128]. However, as previously discussed, PD patients develop a slowly progressive neuropathology that lasts for decades across the premotor and motor symptomatic phases [2,129]. The chronic and changing nature of brain diseases is a critical consideration for small RNA therapeutics, to provide an effective and durable concentration in brain areas may require dosing adjustments over time, and duration of treatment many times longer than those for which the existing technologies have been employed to date.

Chemical modification in RNAi oligonucleotides is a common strategy to produce more specific and sustainable effects [130,131,132,133]. Locked nucleic acids in ribose and phosphorothioate linkages between nucleotides have brought enhanced target specificity and higher nuclease resistance, respectively [134,135]. Both changes were incorporated in miravirsen, the first miRNA-based drug to enter clinical trials, and provided long-term and dose-dependent anti-HCV effects [136,137,138,139]. In addition to miravirsen, the first and second FDA-approved RNAi drugs, patisiran and givosiran, also contain chemically modified nucleotides. Patisiran incorporated oligonucleotides with a 2′-methoxy group on riboses to improve stability in serum, a change incorporated in other therapeutic siRNAs [130,133,135,140]. Givosiran has a modified ribose with either 2′-deoxy-2′-fluoro or 2′-O-methyl, like other siRNAs developed by Alnylam [141,142,143,144,145]. In addition, the drug presents phosphorothioate linkages at the 5′-end of both siRNA strands to prevent nuclease digestion after subcutaneous injection [146,147].

The delivery strategy of oligonucleotides used for brain diseases has been a matter of great importance in RNAi biotechnology. The blood–brain barrier represents a critical obstacle for systemically-injected drugs reaching areas inside the central nervous system [148,149,150]. Some strategies have improved brain penetrance, such as conjugating liposomes with antibodies for delivering plasmid vectors [151]. Indeed, aptamers and monoclonal antibodies are viable molecules for association with RNAi duplexes for site-specific drug delivery [152], as revised elsewhere [153,154,155,156,157,158,159]

Therapeutic small RNAs can be administered locally into brain tissues by specific devices or stereotaxic surgery, thus circumventing the blood–brain barrier. Convection-enhanced delivery (CED) has been experimentally used to inject siRNAs and microRNAs into cerebral areas where brain tumors grow [160,161,162,163,164]. Previous work showed that CED was viable for delivering nanoparticulated RNA duplexes against brain gliomas [161,165,166], a technology potentially exploitable to neurodegenerative diseases.

Stereotaxic surgery is another method to inject oligonucleotides into the brain, providing two main advantages: access to specific regions involved in disease pathogenesis and the bypass of blood–brain barrier. In such sense, new particles and methods have been developed to internalize oligonucleotides inside neurons [167,168].

Magnetic particles, along with magnetofection technology, has brought improvement in the delivery of oligonucleotides [169,170]. Our group recently tested a magnetic carrier, NeuroMag^®^, constituted by polystyrene copolymers coated with iron. This was first used in vivo for transfecting plasmids into pyramidal cells of the rat visual cortex [171]. This particle also shows ability to carry functional microRNA inhibitors to the rat striatal neurons when injected via intracerebroventricular route of administration, causing a significant change in miR-134 content in striatum [172]. In the following section, we will present some recently developed nanoparticles that provide remarkable inputs for miRNA-targeted therapeutics applied to neurodegenerative disorders.

### 5.3. Nanoparticles for microRNA Delivery

First, any miRNA-based therapy is aimed to return aberrantly expressed miRNAs to their physiological level, either by administering synthetic double-stranded RNAs that imitate them, the miRNA mimics, or those that reduce their content, the antisense oligos named anti-miRs [34,173]. To perform their function, these molecules must be first internalized into the cells, which requires some ability to penetrate cell membranes. Transfection is usually difficult due to the high molecular weight and high negative charge of oligonucleotides, leading them to be electrostatically repealed by anionic charges of the cell membrane [174]. Therefore, they commonly cross the lipid bilayer cloned using one of two main strategies, inside a virus genome or complexed with nanoparticles that facilitate their endocytosis [154].

Both viral and non-viral approaches have been widely used in the treatment of cancer [175,176], and many new applications have been reported for neurological diseases, including PD [176,177]. Actually, viral vectors are especially suitable for neurological diseases because they have high transfection efficiency in neurons. Different viral vectors have been used in animal models to deliver miRNAs that regulate genes involved in PD pathogenesis. To mention two examples, (i) a recombinant adeno-associated virus (rAAV) was injected in the mouse brain to overexpress miR-134, in order to provide evidence for a negative role of miR-134 in dendritic arborization of cortical layer V pyramidal neurons in vivo [178]; (ii) injections of lentivirus that overexpresses miR-126 were able to protect brain cells against 6-OHDA injury [54]. Lentivirus combined with siRNAs were effective to treat other cerebral pathology, focal cerebral ischemia. The study showed that microRNA-210 can increase microvascular density and the number of neural progenitors in ischemic brains in mice [179]. To date, several treatments based on viral vectors have entered pre-clinical and clinical trials, several of them to treat PD [180,181,182]. However, viral vectors still hold some risk factors, including immunogenicity, oncogenic transformation [183], off-target effects, as well as other biotechnological limitations like high costs for production, limiting gene packing capacity and difficulties for large scale production [184].

In order to avoid these limitations, research on non-viral vectors emerged as a safer and more affordable alternative. Nanoparticles have the ability to protect RNAi oligos against degradation by nucleases in the serum, and favor their penetration into target cells. Among the advantages of nanoparticles, are the ease of production, reproducibility, ease of storage, low toxicity and immunogenicity, and, in addition, most are biodegradable and biocompatible [142,185,186]. Numerous studies have demonstrated the efficiency of miRNA release through non-viral vectors, mainly in the cancer field. The most widely used transporting nanoparticles contain lipid compounds, polymers and inorganic molecules composed of gold, silica, calcium phosphate and iron oxides [187].

#### 5.3.1. Lipid-Based Particles

Lipid particles containing cationic lipids have been extensively used in experimental research and biotechnological development. They are formed by one or several lipid bilayers between which the nucleic acid is trapped. Cationic lipids have a head with a positively charged amino group that interacts with the negative charges of nucleic acids for complexing and internalization. In addition, the positive charges help the particle to interact with the negatively charged membranes which aids cell transfection. Cationic liposomes can release both DNA [188,189] and RNA constructs [190,191]. In the case of miRNAs, once the nanoparticles loaded with miRNA are successfully internalized into the target cells, they need to avoid the endosomal pathway [192,193] for releasing viable miRNA into cytoplasm and avoid degradation of genetic material [194]. Many studies have generated lipid particles with improved capacity to deliver miRNAs, as reviewed elsewhere [195].

Finally, lipid exosomes can be employed for RNAi delivery [196]. Exosomes are small vesicles formed by lipid bilayers (40 to 100 nm) that carry biological molecules, including mRNAs and miRNAs, playing a role in cell signaling. These small lipid particles have already been used to release siRNAs as therapeutic molecules in numerous studies [196], which include pre-clinical applications for neurodegenerative diseases such as Alzheimer’s [197] and PD [198].

#### 5.3.2. Polymeric Particles

Polymeric carriers are formed by synthetic or natural compounds. Several types of synthetic polymers have been successfully used for carrying miRNAs. Polymers based on poly (ethyleneamine)s (PEIs) provide efficient encapsulation and have demonstrated an ability to carry miR-145 and miR-33a [199,200]. Although a large number of positive charges and non-biodegradability of polymeric carriers can reduce their viability and use, several studies have shown efficacy in PD. Thus, an in vivo study in mice using PEI/siRNA complexes decreased the expression of α-synuclein mRNA and protein in the striatum by about 50% [201] without causing toxic signs. Likewise, several in vitro studies have demonstrated the efficiency of PEG/PEI complexes as vectors for delivering of anti-α-Syn siRNAs to PC12 cells [202].

Poly(lactic-co-glycolic acid) (PLGA) polymers have advantages regarding biocompatibility and biodegradability. They are difficult to load with miRNAs due to their negative charge [203], but they combination with cationic polymers can overcome this limitation. PLGA nanoparticles coated with protamine sulfate are useful to carry miR-124; when injected in the mice brain, the treatment improved the motor signs in 6-OHDA-injured mice model [204]. PLGA particles can also be combined with a rabies virus glycoprotein RVG29 to enhance the transfection of miR-124 into cells of the substantia nigra, after injected by intraventricular route. This miRNA caused neuroprotection, by reducing pro-inflammatory cytokines and cell apoptosis [205].

Polyurethane based particles (PU-based NPs) are carriers with biocompatibility [206] and ability to inhibit brain tumor cells [207].

Natural polymers such as chitosan and hyaluronic acid, are highly biocompatible particles [208,209]. As these particles present a high positive charge that allows efficient RNA loading [210], they present, in contrast, difficulty to release the RNA oligos from the complex. Even so, some groups have managed to use hyaluronic acid-based nanoparticles combined with chitosan, for carrying miRNA for breast cancer therapy [211]. To improve the release, they have been complexed with negative polymers like PLGA [212,213,214].

#### 5.3.3. Inorganic Particles

Inorganic particles possess several of the desirable characteristics for nucleic acid transportation, such as biocompatibility, ease of manufacture and storage, as well as ease of controlling their synthesis. To date, numerous studies have been carried out with this type of inorganic particles such as magnetic particles for miRNA release with different applications like cancer [215] and PD [172,216,217]. Other inorganic carriers, including silica nanoparticles and mesoporous silica have successfully release anti-miRs [218] or miRNA mimics [219] to treat brain cancers.

## 6. Neuroprotective miRNA-Based Approaches in Models of PD

Mounting evidence stands that changes in miRNA content by synthetic oligos could modify the underlying mechanisms of neurodegenerative diseases, as accessed by different experimental studies. The seminal work showing the value of microRNAs as a disease-modifying strategy used a model of temporal lobe epilepsy. Prof. Henshall and collaborators showed that antisense oligonucleotides (antimiRs) targeting miR-134 injected in mice brain ventricles could reduce the loss of hippocampal cells and the number of seizures induced by intra-amygdalar injection of kainic acid [220]. AntimiR-134 was also neuroprotective in multiple models of epilepsy, including pilocarpine, pentylenetetrazol, and stimulation of the perforant pathway [221,222]. Beyond evidencing that microRNAs play a role in the pathogenesis of a degenerative disorder, this ‘proof-of-concept’ work revealed that changing the content of specific microRNAs is a strategy to modify underlying mechanisms of neurodegeneration. Studies regarding PD also evidenced that microRNAs play a role in disease mechanisms and are promising targets for neuroprotection.

A significant decrease in miR-7 was found in the substantia nigra of patients with PD, and, therefore, this change was proposed to play a role in α-Syn accumulation [86]. The neuroprotective actions of miR-7 were first studied in cellular models of PD, which evidenced the ability of this miRNA to target and down-regulate α-Syn by binding to its 3’-UTR mRNA region [36,37]. In agreement, the expression of miR-7 in different tissues during neuronal development is inversely related to the α-Syn content, suggesting that this miRNA exerts a tuning role on α-Syn levels [37]. While the loss of miR-7 function contributes to α-Syn accumulation—which can be reversed by a microRNA replacement, this target also provides additional effects by regulating other genes related to PD pathogenesis [34,36,86]. miR-7 protects cells against oxidative stress by downregulating VDAC1 and Keap1. It also reduces the expression of genes related to the pro-inflammatory process (RelA and NRLP3) and cell glycolysis (RelA), among others, as discussed below [36,38,47,51].

The dysfunction of mitochondrial activity plays a critical role in underlying mechanisms of PD [223,224]. The membrane potential of this organelle is maintained by a complex of proteins named mitochondrial permeability transition pore (mPTP) [225]. However, an excessive opening of the mPTP pores leads to cell death [226,227]. A recent study showed that miR-7 regulates a component of mPTP, the voltage-dependent anion channel 1 (VDAC1) [51]. In MPP+ injured SH-SY5Y cells and primary cortical neurons, overexpression of miR-7 decreased the opening of mPTP channels, which reduced mitochondrial fragmentation/depolarization and mitigated oxidative stress and the release of pro-apoptotic proteins and calcium to the cytoplasm [51]. Also, a previous work using A53T α-Syn parkinsonian mice showed that VDAC1 affects the deposition of this mutant α-Syn in the brainstem, striatum, and cortex, and also induces the opening of mPTP pores [227]. By targeting distinct genes, VDAC1 and α-Syn, miR-7 causes a convergent effect against α-Syn accumulation [36,37,51].

MiR-7 also mitigates oxidative stress by silencing Kelch-like ECH-associated protein 1 (Keap1), a cytoplasmic inhibitory protein. In physiological conditions, Keap1 is found complexed with the erythroid 2–related factor 2 (Nrf2) [228], a nuclear factor that triggers the expression of antioxidant genes [40,229]. The movement of Nrf2 to the cell nucleus is inhibited by Keap1 [230]. In such sense, a miR-7 suppression over Keap1 will consequently allow Nrf2 to translocate to the nucleus for upregulating genes that alleviate oxidative stress. Indeed, overexpression of miR-7 increases glutathione levels causing a 50% decrease of hydroperoxides in MPP+-injured SH-SY5Y cells [40]. In summary, this work added valuable information regarding the cell pathways related to the ‘antioxidant’ effects of miR-7.

Regarding RelA gene, the protective role of miR-7 was also investigated in the MPP+ model [38]. First, miR-7 downregulates RelA, which is a component of the nuclear factor-κB (NF-κB). NF-κB is a pleiotropic transcriptional factor for signaling molecules such as chemokines, cytokines, pro-inflammatory enzymes, adhesion molecules, and pro-inflammatory transcription factors, that ultimately protect neuron cells by orchestrating inflammatory reactions [231]. RelA plays a role in the death of neuron cells exposed to MPP+, and the respective knocking-down by miR-7 caused neuroprotection [38]. Cell glycolysis was also improved by miR-7-induced RelA depletion [51]. Replacement of miR-7 in MPP+-injured SH-SH5Y cells ameliorated glycolysis, with a positive increase in ATP/ADP ratio, glucose consumption, as well as lactic acid production. Indeed, the suppressive action of miR-7 on RelA upregulates the content of surface glucose transporter (Glut3) that also causes a glycolysis-promoting effect. These findings were confirmed by the silencing of the Glut3 gene or through inhibition of the hexokinase enzyme that catalyzes the first step in glycolysis [51].

Neuroinflammation is a component of the neurodegenerative process in PD that is affected by microRNAs [96,232]. The inflammasome protein NRLP3 is a message expressed in microglial cells directly regulated by miR-7. This protein was found activated in the serum of PD patients and also in midbrain regions of parkinsonian mice [47]. Intra-striatal injection of miR-7 mimics in MPTP-induced parkinsonian mice reduces the dopaminergic degeneration and inhibits microglial activation. In addition, the downregulation of NLRP3 inflammasome by miR-7 decreased the content of a pro-inflammatory cytokine, IL-1β. Injection of miR-7 mimics into the striatum also causes neuroprotective results in transgenic A53Ttg/tg parkinsonian mice, corroborating the results found in the MPTP model. Western blotting analysis confirmed that miR-7 down-regulates NLRP3 in mice midbrain, and also inhibits the activation of caspase-1 and decreases IL-1β production [47].

Finally, the microRNA Let-7 also plays a role in α-Syn regulation, affecting an autophagy-lysosome pathway that executes the clearance of α-Syn in healthy cells [87]. The role of let-7 in α-Syn accumulation was studied in a transgenic *C. elegans* model of PD that expresses human α-Syn. Inhibition of let-7 decreased α-Syn expression without affecting dopaminergic/acetylcholinergic neurons. The authors suggested this decrease in α-Syn was related to autophagy and induction of the daf-16 forkhead box O (FOXO) transcription factor that is involved in PD pathogenesis [233]. Igg-1 and atg were also increased in let-7 silenced cells. Finally, let-7 regulates genes that trigger cell apoptosis. Inhibition of let-7 protected cells from apoptosis by reducing two pro-apoptotic genes, ced-4 and jnk-1. Altogether, these results highlight let-7 as a promising miRNA target for PD therapeutics that act against multiple underlying mechanisms of the disease [87].

## 7. Summary and Perspectives

Translating a scientific discovery to the clinics represents a long time and challenging process that require huge efforts for planning and executing many assays, resilience, and money. The first FDA-approval of a small-interfering RNA (siRNA) drug was in 2018, twenty years after the seminal work of Fire & Mello has been published. When siRNA technology becomes available, the scientific community feels a disproportional excitement. Since any gene could be silenced, all diseases related to overexpressed genes would be treatable. However, limitations in siRNA technology rapidly appear, and the excitement gives place to disappointment. The siRNA’s effects lasted for a few days, some off-target effects emerged, and ribonuclease enzymes quickly cleaved RNA duplexes. The first lipid nanoparticles became widely available, the conventional liposomes that facilitated cell transfection. While this ‘first-generation’ liposomes offered protection for RNA molecules, they suffered rapid phagocytosis by circulating macrophages before reaching the target organs. So, only topic preparations were promising, and siRNAs for ophthalmic diseases were the leading drugs for an RNAi market yet in its infancy.

A massive investment of time and money, fortunately, has changed the scenario for the best. New nanoparticles were developed to escape phagocytosis in the blood and to deliver siRNAs safety inside the cell cytoplasm, either lipid or polymeric. Target delivery to specific organs is a reality today. The FDA-approved drugs, patisiran, and givosiran that are administered by intravenous or subcutaneous routes, are efficiently vectorized to the liver for transfecting hepatocytes with siRNAs. Furthermore, new chemical modifications in nucleic acid moieties have provided more specific and long-lasting effects.

Nowadays, several products with RNA interference applications are found in phase II-III clinical trials [130]. Although only a few gene therapy drugs are available in the market for human use, the number of products under clinical trials has increased in the last few years. This increment has augmented the interest of biotechnology companies in developing gene therapy-based drugs and, consequently, to obtain safer and more efficient genetic delivery systems, including miRNAs.

As already mentioned, miRNA mimics and AntimiRs have received biotechnological inputs to enhance the stability of RNA molecules and can be used in combination with nanoparticles with high transfection efficiencies [185]. This progress in nanotechnology aligned with improved changes in oligonucleotide chemistry has provided a notorious evolution in RNAi therapeutics [131,234,235]. Targeted delivery, reduced toxicity, and cost, are issues to be addressed in the future for even better use of short RNA nucleotides [134,236,237,238,239].

Finally, the treatment of brain diseases with small RNAs holds an additional challenge due to the blood–brain barrier. Stereotaxic surgery seems to be the most viable strategy to overcome this obstacle. Although laborious and requiring hospitalization, we could envisage benefits in this procedure, including a tissue-specific delivery that reduces the cost of treatment and avoids effects in other tissues. Moreover, as microRNAs act over different mRNAs, a more widespread effect is achieved and may counteract different underlying mechanisms of PD. In summary, we concluded that miRNAs are promising drug targets for modifying the progressive nature of PD, and future technological inputs will allow translating these small RNAs to the clinics in the coming future.

## Figures and Tables

**Table 1:** Selected microRNAs and related targets involved in PD pathogenesis.

MicroRNA	Target	Indirect Target	Reference
**α-synuclein aggregation**			
miR-7, miR-153, miR34b/c, miR-214	α-SYN		Junn et al., 2009 [36]; Doxakis 2010 [37]; Choi et al., 2014 [38]; Fragkouli & Doxakis 2014 [39]; Kabaria et al., 2015 [40]; Wang et al., 2015 [41]
Let-7		α-SYN	Kumar et al., 2017 [42]
miR-16-1	HSP70	α-SYN	Zhang & Cheng 2014 [43]
**Clearance of α-Syn**			
hsa-miR-21 hsa-miR-224; hsa-miR-373; and hsa-miR-379	LAMP2a		Alvarez-Erviti et al., 2013 [44]
hsa-miR-26b; hsa-miR-106a; and hsa-miR-301b	HSP70		Alvarez-Erviti et al., 2013 [44]
miR-128	TFEB		Decressac et al., 2013 [45]
miR-15b-5p	SIAH1		Chen et al., 2018 [46]
**Neuroinflammation**			
miR-7	NLRP3		Zhou et al., 2016 [47]
miR-155		FADD, SOC1, IKK, IL13Rα1	Ponomarev et al., 2013 [48]
miR-27	ATP5G3		Prajapati et al., 2015 [49]
miR-7116-5p		TNF-α	He et al., 2017 [50]
**Mitocondrial function and Oxidative stress**			
miR-7	VDAC1; KEAP1		Chaudhuri et al., 2016 [51]; Kabaria et al., 2015 [40]
**Apoptosis**			
miR-7	BAX; SIR2		Li et al., 2016 [52]
**Glycolitic pathway and gene expression**			
miR-7	RELA	GLUT3; NFκB	Choi et al., 2014 [38]; Chaudhuri et al., 2015 [51]
**Other pathways**			
miR-124		calpain/cdk5 pathway proteins	Kanagaraj et al., 2014 [53]
miR-126		IGF-1/PI3K/AKT signaling	Kim et al., 2014 [54]
**PRKN and PARK7-Oxidative stress in autosomal recessive parkinsonism**			
miR34b/c		PRKN and PARK7	Miñones-Moyano et al., 2011 [55]
miR-494; miR-4639-5P	PARK7		Xiong et al., 2014 [55]; Chen et al., 2017 [56]
**LRRK2 related**			
miR-205; miR-138-2-3p	PARK8		Cho et al., 2013 [57]; Cardo et al, 2014 [58]
Patogenic LRRK2 inhibit let-7 and miR-184; Let-7 and miR-184 regulate E2f1 and DP, respectively, protecting the cells.	E2f1; DP		Gehrke et al., 2010 [59]

## Supplementary Materials

The following are available online at https://www.mdpi.com/2073-4409/9/4/841/s1, Box 1: Basic definitions in RNA interference, Box 2. FDA guidelines for drug development process applied to miRNA-based drugs.
