# Supplementary material for: The Promise and Challenges of Developing miRNA-Based Therapeutics for Parkinson’s Disease
Source: Cells. 2020 Mar 31;9(4):841. doi: 10.3390/cells9040841 (PMC7226753; doi:10.3390/cells9040841)
Supplement: Supplementary file 1 [file cells-09-00841-s001.pdf]

Box 1. Basic definitions in RNA interference

RNA interference (RNAi)
The term RNAi refers to a process of gene knock-down that occurs at the post-transcriptional level, thus affecting the content of expressed genes. The process is triggered by short RNA molecules, either small-interfering RNAs or microRNAs (see definitions below), and finally results in the degradation of mRNAs bearing complementary sequences.

Small-interfering RNA (siRNA)
siRNAs are short double-stranded RNAs containing ≈ 22 nucleotides. These duplexes engage a complex of proteins named RISC (RNA-induced silencing complex) responsible for mRNA cleavage. siRNAs are commonly used to knock-down one unique gene of interest and are commercially available.

MicroRNA (miRNA)
The term microRNA refers to biological molecules that, after processing, will present a structure similar to a siRNA, i.e., a short double-stranded structure RNA molecule. miRNAs also recruit the RISC complex for gene silencing, but they bind to hundreds of mRNAs bearing partially complementary sequences at 3'-untranslated (3'-UTR) region.

miRNA mimics
A microRNA imitator, composed by short double-stranded RNA nucleotides identical to the endogenous miRNA. Synthetic miRNA mimics are used in experimental assays to generate a condition in which the endogenous microRNA is present at the physiological level or overexpressed.

AntimiR
AntimiRs are microRNA inhibitors, formed by a single-stranded chain of antisense nucleotides. The antimiR will bind to a specific endogenous mature miRNA containing a complementary sequence, thus blocking the miRNA function in the cell.

Box 2. FDA guidelines for drug development process applied to miRNA-based drugs.

The following information describes how the conventional process of drug discovery and testing could be applied for miRNA-based drugs, as shown in figure 1. In the discovery stage, **Step 1**, researchers will test if a candidate miRNA target has the potential to produce therapeutic effects. In PD, for example, a synthetic oligonucleotide that inhibits or, in contrast, imitates a specific microRNA must show efficacy to protect neurons exposed to a damaging agent. Researchers must employ experimental preparations that recapitulate changes found in the parkinsonian brain – the models of PD. Preliminary analysis *in silico* may inform if the candidate microRNA is involved in underlying mechanisms of the disease, for example, regulating pathogenic proteins like α-Syn. Finally, promising targets are those microRNAs with aberrant patterns of expression in PD patients and animal models. In the subsequent

‘development’ phase of Step 1, the study may incorporate technologies in the candidate miRNA-based drug. Which is the best nanoparticle for brain delivery? Should the microRNA nucleotides receive chemical modifications to improve specificity and durability? How to inject the preparation for reaching affected brain areas? Which is the lowest dose of oligonucleotides that protect neurons without causing toxicity?

The candidate miRNA-based drug discovered in Step 1 will progress to **Step 2** for preclinical testing of efficacy and safety, before entering the clinical research. A microRNA inhibitor for hepatitis C named miravirsen is a good example of drug discovery/development and also preclinical testing in non-human primates, as described by Lindow & Kaupinen et al. (2012) [1]. Pre-clinical testing of anti-Parkinson drugs requires a model that best recapitulates critical features of PD (i.e., the loss of nigral cells, alpha-synuclein accumulation, motor deficit) and that provides reliable endpoints of neuroprotection and motor improvement, as reviewed elsewhere [2].

Clinical research, **Step 3,** encompasses three phases that explore how the candidate drug acts in the human body. The number of individuals, cost, and duration of the study increase progressively as the drug reaches endpoints and evolves to the subsequent phase. Phase I trials addresses safety and dosage. The study normally recruits healthy volunteers (N=20 – 100) but may also involve patients with the disease/condition to which the drug was developed. Phase I studies commonly execute a dose escalation analysis to find the maximum tolerated dose and explore pharmacodynamic and pharmacokinetic properties. In Phase II, efficacy and side effects are examined in a higher number of individuals (N=50 – 500) that present the disease/condition. Finally, Phase III clinical research is a multicentric and multi-country trial aimed to test if the drug shows efficacy in an even larger sample of individuals who have the disease (N=300 – 3,000) and causes no significant adverse effects. A previous review illustrated the road of selected RNAi-based drugs across Phases I – III that finally led to the first FDA-approved small-interfering RNAs (siRNAs), patisiran and givosiran in 2018 and 2019, respectively [3,4]. Brief definitions of RNAi, siRNA, miRNA mimics, and AntimiR are present in Box 1 of supplementary material.

In **Step IV**, all data have been produced across clinical trials, especially the Phase III, and FDA will examine if the drug is safe and effective for the intended disease. A critical decision on whether to approve or not the drug is based on a document named the ‘new drug application’ (NDA) that contains all results generated across the development process. Prescribing information is commonly refined at this stage, referred as the ‘labeling’ process. FDA decision at Step 4 is the final part of product development, and the approved drug is allowed to entering the market. The FDA-approved drug is now ready for commercialization and use for the intended disease. Currently, two RNAi-based drugs received FDA approval: patisiran [4], and givosiran [5].

Post-marketing safety monitoring represent the **Step 5** of drug development, commonly refereed as the pharmacovigilance study or Phase IV. FDA keeps monitoring drugs in the marketplace, to check if some undesirable effect has emerged and deserves some action. If safety issues appear when drug usage reaches a large number of individuals, FDA will adopt appropriate measures regarding cautions on dosage or usage information, or, indeed, some more restrictive actions.

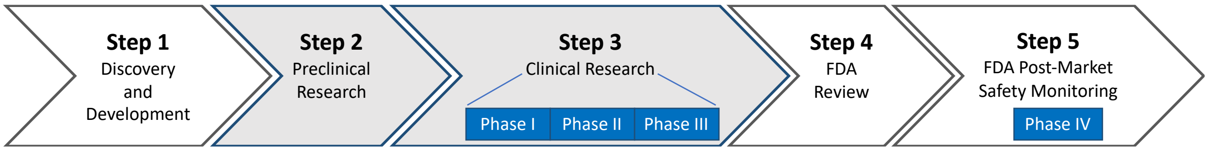

Figure 1. Multi-step development of a biotechnological product. The process begins at the stage of discovery and development (step 1), with posterior preclinical testing in animals (step 2), and the subsequent clinical trials (Phase I - III). FDA review and approval in Step IV allows the drug to enter the market. A subsequent Step 5 represents the pharmacovigilance phase for FDA-approved drugs.

References

1. Lindow, M.; Kauppinen, S. Discovering the first microRNA-targeted drug. *J Cell Biol* **2012**, *199*, 407-412, doi:10.1083/jcb.201208082.
2. Koprich, J.B.; Kalia, L.V.; Brotchie, J.M. Animal models of alpha-synucleinopathy for Parkinson disease drug development. *Nat Rev Neurosci* **2017**, *18*, 515-529, doi:10.1038/nrn.2017.75.
3. Titze-de-Almeida, R.; David, C.; Titze-de-Almeida, S.S. The Race of 10 Synthetic RNAi-Based Drugs to the Pharmaceutical Market. *Pharm Res* **2017**, *34*, 1339-1363, doi:10.1007/s11095-017-2134-2.
4. Titze-de-Almeida, S.S.; Brandao, P.R.P.; Faber, I.; Titze-de-Almeida, R. Leading RNA Interference Therapeutics Part 1: Silencing Hereditary Transthyretin Amyloidosis, with a Focus on Patisiran. *Mol Diagn Ther* **2019**, 10.1007/s40291-019-00434-w, doi:10.1007/s40291-019-00434-w.
5. de Paula Brandao, P.R.; Titze-de-Almeida, S.S.; Titze-de-Almeida, R. Leading RNA Interference Therapeutics Part 2: Silencing Delta-Aminolevulinic Acid Synthase 1, with a Focus on Givosiran. *Mol Diagn Ther* **2019**, 10.1007/s40291-019-00438-6, doi:10.1007/s40291-019-00438-6.
